# The VraSR regulatory system contributes to virulence in Streptococcus suis via resistance to innate immune defenses

**DOI:** 10.1080/21505594.2018.1430466

**Published:** 2018-03-19

**Authors:** Nadya Velikova

**Affiliations:** Host-microbe Interactomics Group, Animal Sciences Department, Wageningen University, De Elst 1, Wageningen, The Netherlands

**Keywords:** antibacterial drug targets, Streptococcus suis, swine industry, two-component systems, virulence, zoonotic pathogens

*Streptococcus suis* is a microbe commonly present in pigs in many parts of the world. Usually *S. suis* does not cause disease neither in pigs, nor in humans. However, a number of outbreaks of *S. suis* have been reported for the last two decades and have raised the concern about zoonotic infections via infected pork meat. The human disease has mostly a zoonotic and occupational origin, however, in the Southeast Asia most people become infected by habitual consumption of raw or undercooked pork, blood and offal products in the form of traditional dishes. Human infections can be prevented by appropriate personal protective equipment and avoiding raw pork or pig blood. However, *S. suis* infections lead to considerable economical losses to the swine industry around the world. The estimated losses only in the USA are approximately USD 300 million annually [1]. Taken all together, these justify the need of research on *S. suis* [2].

More than 30 serotypes of *S. suis* have been identified based on the antigenic diversity of the capsular polysaccharide, a virulence factor of different *S. suis* serotypes [3]. So far, serotype 2 is the most prevalent type in humans infected with *S. suis* [4]. It is known that not all serotype 2 strains are virulent, and the degree of virulence varies among strains [5].

Chang et al. have studied the role of two-component systems (TCS) in the interaction between *S. suis* 2 and the innate immune response *in vitro* and *in vivo* [6]. TCS are the major signalling systems in bacteria and they are involved, among others, in the regulation of virulence in many pathogens [7,8]. This is one of the reasons why TCS are considered and have been explored as promising targets for antibacterial therapy [9,10]. The antibacterial effect of putative TCS inhibitors against *S. suis in vitro*, more specifically putative histidine-kinase autophosphorylation inhibitors, have been recently reported [11]. Considering the growing problem of antibiotic resistance and the anticipated resistance development to bactericidal agents, targeting virulence, including TCS signalling modulation, has been considered a promising alternative approach for therapeutics with lower potential of resistance development [12]. Chang et al. reported that several response regulators of *S. suis* 2 were significantly up-regulated following stimulation with polymorphonuclear leukocytes (PMNs), providing evidence that that *S. suis* 2 rely on multiple TCS to survive during host infection. The involved TCS include CiaRH which is known to contribute to *S. suis* 2 virulence *in vivo* [13,14,15], the homologue of YycFG, which is essential for the viability of low GC-content Gram-positive pathogens [16,17], and a novel TCS, the VraSR, which Chan et al. have extensively studied thereafter. Bioinformatics research revealed that VraRS is fairly conserved among 25 *S. suis* strains with different serotypes and genotypes. The authors observed difference in the thickness of the polysaccharide capsule between the capsule of the wt *S. suis* 2 and the constructed mutant strain lacking VraSR (Δ*vraSR*), suggesting that VraRS might contribute to the regulation of the thickness of the polysaccharide capsule, and thus contribute to virulence. Evidence is provided that VraSR contributes to the survival of *S. suis* 2 in human blood *in vitro* by promoting resistance to and evading innate immune responses. Furthermore, Chang et al. report that VraSR promotes *S. suis* 2 adhesion *in vitro*, possibly contributing to its ability to pass the blood brain barrier and to cause meningitides. Last but not least, the study provides evidence that VraSR contributes to *S. suis* 2 virulence *in vivo*. Altogether, the reported data sheds light on the role of VraSR in S. suis 2 resistance to the host innate immune response and in the virulence of *S. suis* 2.

The study provides a foundation for further research in the precise molecular mechanisms underlying VraSR-regulated virulence and immune evasion by *S. suis* 2 and opens up the question of the role of VraSR-signalling in other *S. suis* serotypes and of VraSR homologues in other pathogens. Furthermore, the reported results suggest that VraSR signalling modulation can serve as a target for antivirulence and antibacteiral therapy against *S. suis* 2.
